# The Bone Lid Technique in Oral and Maxillofacial Surgery: A Scoping Review

**DOI:** 10.3390/jcm11133667

**Published:** 2022-06-24

**Authors:** Stefano Sivolella, Giulia Brunello, Sourav Panda, Lucia Schiavon, Fouad Khoury, Massimo Del Fabbro

**Affiliations:** 1 Department of Neurosciences, Dentistry Section, University of Padova, Via Giustiniani 2, 35128 Padova, Italy; giulia-bru@libero.it (G.B.); luciaschiavon.08@gmail.com (L.S.); 2 Department of Oral Surgery, University Clinic of Düsseldorf, Moorenstr. 5, 40225 Düsseldorf, Germany; 3 Department of Biomedical, Surgical and Dental Sciences, Università degli Studi di Milano, Via Commenda 10, 20122 Milan, Italy; drsaurav87@gmail.com (S.P.); massimo.delfabbro@unimi.it (M.D.F.); 4 Department of Periodontics and Oral Implantology, Institute of Dental Sciences, Siksha O Anusandhan University, Bhubaneswar, Odisha 751003, India; 5 Department of Oral and Maxillofacial Surgery, University of Munster, Waldeyerstr. 30, 48149 Munster, Germany; prof.khoury@t-online.de; 6 Private Clinic Schloss Schellenstein, International Dental Implant Center, Am Schellenstein 1, 59939 Olsberg, Germany; 7 I.R.C.C.S. Orthopedic Institute Galeazzi, Via Galeazzi 4, 20161 Milan, Italy

**Keywords:** bone lid, bone window, bone defect, oral surgery, maxillary sinus, cyst, impacted tooth, apicoectomy

## Abstract

This scoping review aimed at reporting the outcomes of the bone lid technique in oral surgery in terms of bone healing, ridge preservation, and incidence of complications. Bone-cutting instruments and stabilization methods were also considered. PubMed, Scopus, and the Cochrane Register of Controlled Trials were searched using a combination of terms, including bone lid, bony window, piezosurgery, microsaw, cysts, endodontic surgery, impacted teeth, and maxillary sinus. A hand search was also performed. The last search was conducted on 30 November 2021. No date limitation was set. Searches were restricted to human clinical studies published in English. All types of study design were considered except reviews and case reports. After a two-step evaluation, 20 (2 randomized studies, 2 case-control studies, 3 cohort studies, 13 case series) out of 647 screened studies were included, reporting on 752 bone lid procedures. The bone lid technique was associated with favorable bone healing when compared to other methods, and with a very low incidence of major complications. Clinical indications, surgical procedures, study design, follow-up duration, and outcomes varied among the studies. Overall, favorable outcomes were reported using the bone lid approach, though evidence-based studies were scarce.

## 1. Introduction

The bone lid technique consists of the preparation and removal of a bone lid or window that is replaced in its original position at the end of the surgery. The aims of the technique are to achieve a valid exposure of the surgical target, to save bone otherwise lost with other more aggressive methods (ostectomy), and to improve bone healing.

This technique was firstly described for the closure following opening of the maxillary antrum and for endodontic surgical treatment of lower molars, with good results [1].

The indications for this technique were then extended to the enucleation of cysts [2,3,4] and other benign lesions [5,6]. Other applications include the extraction of deeply fractured roots or impacted teeth [7,8], the removal of fracture or failed implants [9,10], the retrieval of implants accidentally displaced into the mandibular bone marrow space [11], and inferior alveolar nerve lateralization [12]. The use of the bone lid technique is indicated for the treatment of pathologies affecting the maxillary sinus, the removal of displaced foreign bodies [13,14], and to seal the antral lateral wall for sinus lift [15]. A combination with the transnasal endoscopic approach has also been reported for the treatment of maxillary sinus pathologies [16,17].

The surgical area is usually accessed via an intraoral approach. Access to deeply impacted lower molars and large mandibular lesions has also been achieved via a submandibular incision [6,18], whose main drawbacks are the risk of facial nerve injury and aesthetic implications due to skin scars.

Precise and thin osteotomies for outlining the bony window can be obtained with both a disc microsaw [9,10] and piezosurgery [2,4,7]. Other instruments, such as traditional rotary burs [1,19], reciprocating saws [20] oscillating saws [21] or lasers [22], have been reported.

An adequate bone lid thickness, together with an optimized design characterized by thin and beveled osteotomies, facilitates the removal of the bony segment, enables its exact fit at the end of the surgery, and increases the contact area at the bone-to-bone interface, thus reducing the need for any additional means of fixation and favoring bone lid revascularization [9]. In case of lack of stability of the repositioned bone lid, various fixation methods have been described, including transcortical screws [9,23], metal [4,8] and resorbable [7,24] mini-plates and screws, sutures [14,19], wires [3], cyanoacrylate-based surgical glue [20], and resorbable pins [25].

Computed tomography (CT) and digital technologies have been applied in surgical planning, as well as in postoperative outcome assessment [4,26]. In recent years, virtual planning has allowed the design of customized surgical templates, produced by milling or through additive manufacturing, which define precise cutting planes [12,27]. The successful application of a computer-assisted intraoperative navigation system for bone lid surgery has also been reported [28].

To the best of our knowledge there is no comprehensive review on this technique, which is deemed to be of great relevance in oral surgery as a bone-saving approach. As the indications for this technique are multiple, and because we expected to find heterogeneous reports, which would be difficult to summarize into a meta-analysis, we decided to perform a scoping review. The latter, in fact, may “examine the extent, range and nature of the evidence on a topic”, and represents the best choice to “summarize findings from a body of knowledge that is heterogeneous in methods or discipline” [29].

The aim of the present scoping review was to summarize the outcomes of the bone lid technique in oral surgery, in terms of bone healing and ridge preservation, based on published evidence.

## 2. Materials and Methods

The present review was performed and reported according to the Preferred Reporting Items for Systematic Reviews and Meta-Analyses extension for Scoping Reviews (PRISMA-ScR) (http://prisma-statement.org accessed on 30 November 2021). The protocol was registered with the Open Science Framework (OSF).

### 2.1. Search Strategy

An electronic search was performed in the following databases: PubMed, Scopus, and the Cochrane Central Register of Controlled Trials (CENTRAL). The last search was performed on 30 November 2021. Search details are provided in Table 1. Search terms were used alone or in combination using the Boolean operators OR and AND. Furthermore, a hand search of issues up to the last issue available on 30 November 2021, including the “Early view” (or equivalent) section, was undertaken in the following journals: *British Journal of Oral and Maxillofacial Surgery; Clinical Implant Dentistry and Related Research; Clinical Oral Implants Research; Implant Dentistry; International Journal of Oral and Maxillofacial Implants; International Journal of Oral and Maxillofacial Surgery; Journal of Oral and Maxillofacial Surgery; Journal of Oral Implantology;* and *Oral Surgery, Oral Medicine, Oral Pathology, Oral Radiology and Endodontology*. The reference list of the retrieved reviews and of the included studies was also searched for possible additional eligible studies not identified by other search methods.

### 2.2. Inclusion Criteria

To be included, studies had to report clinical results of oral surgery procedures in which the bone lid technique was used to cover and protect the healing site in order to improve the clinical and radiographic outcome.

The search was limited to clinical studies reporting on at least 10 cases of the bone lid technique published in the English language involving human subjects. Both prospective and retrospective studies were included. The studies had to provide details on the type of clinical application, the patients’ selection criteria, the procedure for applying the bone lid, the duration of the follow-up, and the number and type of complications. They also had to provide clear definitions of the clinical and/or radiographic outcomes used to assess the success or failure of the procedure.

Publications that did not meet the above inclusion criteria and those that did not deal with original clinical cases (e.g., reviews and technical reports) were excluded. Papers in which the bone lid technique was applied in combination with maxillary sinus augmentation were excluded. Multiple publications of the same pool of patients were also excluded. When papers from the same group of authors with very similar databases of patients, materials, methods, and outcomes were identified, the authors were contacted to clarify whether the pool of patients was indeed the same. In case of multiple publications relative to consecutive phases of the same study or to enlargements of the original sample size, only the most recent data (those with the longer follow-up and the larger sample size) were considered.

### 2.3. Selection of the Studies

Two reviewers (G.B. and L.S.) independently screened the titles and the abstracts of the articles initially retrieved through the electronic search. The concordance between reviewers was assessed by means of the Cohen’s Kappa coefficient. In case of disagreement, a joint decision was made through discussion, or by consulting a third reviewer (M.D.F.). The full text of all studies of possible relevance were independently assessed by the same two reviewers to check if they met all inclusion criteria. For articles excluded at this stage, the reason for exclusion was recorded. The included studies were divided according to the type of clinical application: endodontic surgery, access to mandibular lesions and impacted teeth, implant explantation, access to the maxillary sinus, and other indications.

### 2.4. Data Charting

Data were extracted by two reviewers independently (G.B. and L.S.). Cases of disagreement were subject to joint evaluation until an agreement was reached. In case of doubts, a third reviewer was consulted (M.D.F.).

The main variables extracted from each included study were the following: study design, sample size, number of surgeons involved; patients’ genders and ages, proportion of smokers, jaw (maxilla or mandible), bone-cutting devices, fixation method, any outcome variable used to evaluate treatment success, follow-up duration, number and type of complications and time they occurred, and the quality of life of patients as well as their satisfaction, as assessed by means of questionnaires or interviews.

The following methodological parameters were also recorded: selection of participants, sample size (the risk of bias was assumed to be low, medium, or high if >50, 10–50, or <10 patients were treated, respectively), length of follow-up period (it was assumed to be low, medium, or high if the mean follow-up duration was >5 years, 1–5 years, or <1 year, respectively), dropouts (it was assumed to be low, medium, or high if dropouts were <5%, 5–15%, or >15%, respectively), measurement of the outcome, and selection of reported results.

The methodological quality of the selected studies was evaluated independently and in duplicate by two reviewers (M.D.F. and S.P.). The tool reported in the *Cochrane Handbook for Systematic Reviews of Interventions*, version 5.1.0 was used for RCTs [30], and a modified ROBINS-I (“Risk Of Bias In Non-randomised Studies—of Interventions”) tool was used for non-randomized studies [31]. All the criteria were assessed as low, moderate (uncertain), or high. The authors of the included studies were contacted to provide clarifications or missing information as needed. Studies were considered to have a low risk of bias (RoB) (green) if more than 2/3 of the parameters were judged as “low” and none as “high”; they were considered to have a moderate RoB (yellow) with 1 to 4 parameters judged as “low” and the rest as “moderate”, with none at high risk. All papers with at least one score at high risk were classified as having high RoB (red).

### 2.5. Synthesis of Results

Descriptive statistics of the included studies were recorded by summarizing the total number of patients and cases treated with the bone lid technique, as well as the postsurgical adverse events for each surgery procedure considered.

## 3. Results

**Figure 1:**
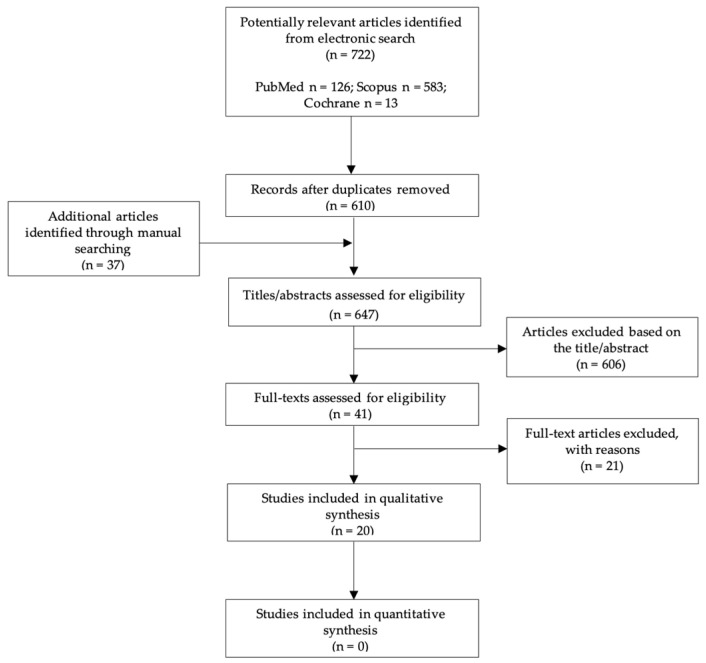
Flowchart of the article selection procedure.

A flowchart summarizing the screening process is presented in Figure 1. The electronic search yielded a total of 610 articles. Thirty-seven additional articles were found by hand-searching. After a first screening of the titles and abstracts, a total of 41 articles reporting results of clinical studies on patients that underwent oral surgery procedures in combination with the use of the bone lid technique were selected. After evaluation of the full-text of these articles, 21 of them were excluded [7,8,13,16,23,24,25,32,33,34,35,36,37,38,39,40,41,42,43,44,45]. The reasons for exclusion are listed in Table 2. A total of 20 studies published in the years 1984–2021 were included. The kappa values for inter-reviewer agreement were 0.95 for both the title/abstract selection and full-text articles, thus indicating an almost perfect agreement between the two independent reviewers. Since a sufficient number of homogeneous studies to be aggregated could not be found, a quantitative analysis was not undertaken.

The main features of the included studies are shown in Table 3 (i.e., study design, clinical indication, sample size, patients’ genders and ages, jaw, number of bone lid cases, follow-up duration, osteotomy technique, and fixation method).

A total of 2 RCTs (reporting on a total of 60 bone lid patients), 2 longitudinal prospective cohort studies (218 bone lid patients), 1 retrospective cohort study (30 bone lid patients), 2 case-control studies (56 bone lid patients), and 13 case series (371 bone lid patients) were found. The included studies reported on a total of 838 patients, of which 735 were treated with the bone lid approach (752 bone lids performed).

The number of clinicians who performed the surgeries was reported in nine studies. In detail, in six studies the surgeries were carried out by a single clinician [2,9,26,47,49,50], whereas only one study involved two operators [14]. One paper reported that five clinicians performed the surgeries [3], while in another study multiple surgeons provided the treatments [56].

Only 2 out of 20 studies provided detailed information on smokers. In a randomized clinical trial comparing piezoelectric surgery to the conventional surgery (rotatory instruments) in mandibular cyst enucleation, only non-smokers were included [2]. In a retrospective cohort study, approximately one third of the included patients were smokers, and smoking habit was found to be a risk factor for bone lid necrosis (*p* = 0.005). Indeed, necrosis was observed only in smokers [46].

In half of the included papers, the surgical procedures were performed under local anesthesia, while in the other three articles, they were also completed in combination with conscious sedation [4,46] or general anesthesia [17,46]. In three studies, the surgeries were carried out either under general or local anesthesia [48,52,54]. The remaining four articles reported no information regarding the type of anesthesia [3,51,53,56].

As shown in Table 3, in all the included papers except one [3], details on the bone-cutting tools were provided. Piezosurgery and microsaws were the most frequently reported cutting methods. Rotary instruments, sometimes in combination with chisels, were also used to create the bony windows. The majority of neuronal complications were reported when disks and burs were utilized [1,25,47,48,54,55], whereas only one case of permanent paresthesia occurred with piezosurgery [4].

The stabilization of the bone lid was achieved without the need of rigid fixation in three studies. Bone lids were stabilized with mini-/microplates and screws in four articles, while the use of resorbable sutures was reported for all cases in four studies in the maxilla. Other fixation methods included resorbable miniplates and screws (one study). In the remaining eight articles, multiple fixation methods were reported. Bone lid resorption, necrosis, and removal were rather infrequent, as only 20 cases were found out of 752, of which 5 were in the maxilla [26,51,53] and 12 were in the mandible [1,3,4,46,48], while it was not specified in 3 cases [9]. In 5 cases out of 17, bone lid complications were clearly associated with suture stabilization or no fixation [1,48,53], 2 with rigid fixation methods [4,26], and 3 with resorbable plates [46]; whereas in 10 cases, the fixation method could not be identified [3,9,51]. In Sukegawa et al., different resorbable plates were utilized, and the osteosynthesis material was not found to be related to the bone lid necrosis [46].

The included studies were divided according to the type of surgical procedure: root-end surgery, access to mandibular lesions and impacted teeth, implant explantation, access to the maxillary sinus, and surgery for other indications. The main results of the included studies are reported in Table 4.

Two studies [1,55] comprising 99 bone lids reported on the use of this technique to perform root-end surgery (apicoectomy), with successful healing except in two cases (Table 4).

Six papers [2,3,4,46,47,48], including 139 bone lids, described the use of this approach to gain access to lesions, such as cysts and impacted teeth in the mandible (Table 4). An RCT comparing the bone lid with piezosurgery versus bone removal with conventional rotary instruments showed a better postoperative recovery in the bone lid group [2]. In Sivolella et al., no cyst recurrence occurred, and in the 11 cases in which it was possible to compare preoperative and 1-year follow-up CT scans, a mean volume reduction of 93.8% in the radiolucent areas was seen [4]. In a retrospective case-control study, complications were registered in 35.3% of bone lid cases, whereas no undesired effects were observed in the not repositioned group [3]. In one of these six articles, an extensive use of customized surgical guides was reported [47].

In two articles, the bone lid technique was described for the explantation of implants owing to peri-implantitis or implant fracture (see Table 4) [9,10]. Compromised implant removal was achieved in all cases, and good healing of the reimplanted bone was obtained in 153 cases out of 156 [9].

In 11 articles comprising 290 bone lids, the clinical indication was maxillary sinus access, either for the treatment of pathologies such as cysts, or for the retrieval of displaced roots or foreign bodies [14,17,26,49,50,51,52,53,54,56]. As evidenced in Table 4, a limited number of permanent complications occurred, and in a few cases, revision surgery, such as for recurrence, was needed.

In a randomized prospective study, the bony window healing was radiographically assessed 3 months after surgery in patients treated either with a bone lid as a free bone graft or with a pedicled bone lid [53]. Better results in terms of bone lid consolidation and bone density, as determined by means of CT scans, were observed in patients who received the pedicled one.

In the remaining cases (Table 4), the bone lid was applied for fractured teeth/roots and foreign body removal (29 cases), impacted tooth extraction (14 cases), inferior alveolar nerve lateralization (4 cases) [9], or for the removal of maxillary lesions [46].

Finally, in none of the included studies were patients’ quality of life and satisfaction assessed by means of questionnaires or interviews.

The RoB summary of the two included RCTs is described in Table 5. Regarding the remaining observational studies (Table 6), the majority of the studies (8 out of 18) were judged at moderate risk of bias, while 6 studies were judged to be at high risk of bias and 4 at low risk of bias.

## 4. Discussion

The present scoping review set few limitations on the sources of evidence (clinical studies with at least 10 participants and English language) in order to give an overview as inclusive as possible of bone lid applications. Despite the existence of heterogeneity and the lack of comparative studies in this field, the results of the present scoping review, which included 752 cases, showed that the bone lid is a feasible and successful technique in various types of oral surgery procedures, and is accompanied by a low incidence of major complications. Indeed, it seems to be a conservative technique that could reduce the amount of bone removal in particular circumstances, such as deeply impacted wisdom teeth, in which otherwise abundant osteotomy would be required. In addition, the success of this technique might be related to the skills and the experience of the operator, and unfortunately none of the included studies analyzed this crucial aspect.

In the largest prospective cohort study included [9], 200 consecutive patients were followed for at least 4 years. The bone lid approach was applied in conservative pre-implant and implant surgery. As a main result, 98.5% of bone lids healed without any complication, ensuring an adequate volume for the planned implant therapy, and limiting the need for supplementary regenerative procedure and bone donor sites. As also reported in Sivolella et al. [4], one of the most important factors outlined by the authors was the bone lid thickness, which is fundamental for its stabilization and revascularization [9,57,58].

When bone lid approach was reported for root-end surgery of lower molars [1,55], better access and intraoperative visibility to the endodontic lesion, associated with a reduced bone removal, were advocated. The remaining bony defect was reduced, allowing a better environment for complete healing. Overall, short-term results seemed to be as good as those reported historically for root-end surgery without a bone lid [59,60]. However, no comparative study was available.

As regards access to mandibular lesions and impacted teeth, one included RCT aimed to compare the use of the bone lid versus bone removal to gain access to alveolar bone lesions [2]. Short-term data on post-operative swelling, immediate neurological complications, and patients’ subjective response to pain were reported. The results supported the superiority of the bone lid technique. The same technique comparison was presented in a case-control study with a longer follow-up [3]. A tendency to adopt the bone lid technique for large-sized mandibular lesions, which might be considered a selection bias, was described. The critical status of those lesions probably justified the higher rate of complications in the bone lid group. It must be noted that both studies mainly focused on the comparison of piezosurgery vs. rotary instruments, rather than on the bone lid technique [2,3].

Improvements to the technique may derive by the use of computer-designed customized cutting guides, which allow for a pre-planned accurate outline of the osteotomies and, subsequently, better lid realignment and stability [47]. This approach may further improve the outcomes of the bone lid technique [12,27,38,47,61], making it more easily applicable as well. This topic should be further investigated with future RCTs comparing free-hand vs computer-guided bone lid surgery.

Partially osseointegrated failed implants (e.g., fractured or affected by peri-implantitis) are usually surgically removed with a trephine bur, thin Lindemann bur, dedicated piezosurgery inserts, or using a reverse high-torque wrench [62]. These methods may be associated with excessive bone loss or operative difficulties. When thin bone plates or deeply located implant body are present, the bone lid technique allows the surgeon to maintain the bone plate and immediately insert a new implant [9,10,63,64]. In pre-implant cases, at the time of delayed implant placement, complete re-osseointegration of the bony window and filling of the bone defect underlying the bone lid also were frequently observed, with no need for bone grafting [4,9].

The bone lid technique, eventually associated with endoscopic sinus surgery [17,41,44,49,50,65], was developed as a conservative method for the closure of the maxillary antrum [13,14,44,57,65,66,67,68]. In order to enhance its re-integration, pedicled bony windows were proposed [14,16,19,52,53,56,66]. The periosteal attachment seems to be beneficial for nourishing the osteotomized bone, as reported in one of the included RCTs [53]. In the present review, only 5 out of 290 bone lids performed to obtain access to the maxillary sinus were lost due to infection [26,50,51,53].

Thin and beveled osteotomy, which can be obtained with both a microsaw and piezosurgery, can provide an ideal self-retentive morphology of the lid, thus reducing the need for rigid fixation [4,9,10,47,53]. Piezosurgery might present potential advantages, including ease of handling and less danger in case of accidental contact with soft tissue [69]. Fixation devices such as microplates, screws, or metallic ligatures represent a stable fixation method, but may have some drawbacks, such as undesired tension on the lid, screw or plate superficialization, and patient complaints [3,4,26,50]. As a consequence, a second surgery for their removal may be necessary.

The recurrence rate was rarely described as a clear outcome [3,4,17,46,48,50]. No recurrence was reported after cyst removal bone lid surgeries. One recurrence was recorded in a study on bone lids associated with functional endoscopic sinus surgery for the treatment of a fungus ball of the maxillary sinus [17].

The results of this scoping review confirmed that the bone lid technique is associated with good outcomes, resulting in a bone-saving approach. However, the results of this review must be interpreted with caution. Among the limitations of the present review, it is worth mentioning the design of the included studies, the variety of instruments utilized, the advancements of the technologies available for both treatment planning and post-op assessment, the different surgical applications, and the short follow-up period of the majority of the studies. Despite that this technique is deemed to be influenced by the experience of the surgeon, its role has not been evaluated. In conclusion, considering the limited number of controlled trials on this topic, the low-quality evidence, and the heterogeneity of the examined clinical studies, randomized clinical trials are needed to assess the effectiveness of the bone lid technique over other approaches. Moreover, which is the most appropriate cutting tool for bone lid fashioning has not been determined so far. Similarly, the usefulness of further fixation methods in cases of well-fitted and stable bone lids is not clear. Virtual planning and the application of customized computer-designed guides might help to improve the outcomes of the technique and its reproducibility.

## Figures and Tables

**Table 1:** Search strategies for the different databases.

Database	Search Strategy
PubMed	((“bone lid” [All Fields] OR “bony lid” [All Fields]) OR “bony window” [All Fields]) AND ((((((((((((((((((((((((((((“methods” [MeSH Subheading] OR “methods” [All Fields]) OR “techniques” [All Fields]) OR “methods”[MeSH Terms]) OR “technique” [All Fields]) OR “technique s” [All Fields]) OR (“piezosurgery” [MeSH Terms] OR “piezosurgery” [All Fields])) OR “microsaw” [All Fields]) OR “micro-saw” [All Fields]) OR “bone defect*” [All Fields]) OR “oral surgery” [All Fields]) OR ((((“maxilla”[MeSH Terms] OR “maxilla” [All Fields]) OR “maxillae” [All Fields]) OR “maxillas”[All Fields]))) OR (((“mandible” [MeSH Terms] OR “mandible” [All Fields]) OR “mandibles”[All Fields]) OR “mandible s” [All Fields])) OR “maxillary sinus” [All Fields]) OR (((“apicoectomy”[MeSH Terms] OR “apicoectomy” [All Fields]) OR “apicectomies” [All Fields]) OR “apicectomy” [All Fields])) OR “apical surgery” [All Fields]) OR “endodontic surgery” [All Fields]) OR “root-end surgery” [All Fields]) OR “implant*” [All Fields]) OR “impacted teeth” [All Fields]) OR “impacted tooth” [All Fields]) OR “impacted molar*” [All Fields]) OR “third molar*” [All Fields]) OR “inferior alveolar nerve*” [All Fields]) OR “cyst*” [All Fields]) OR “cystic lesion*” [All Fields]) OR “computer-guided” [All Fields]) OR “osteoplastic procedure*” [All Fields]) OR “sinus surgeries” [All Fields]) Filter: English
Scopus	(“bone lid” OR “bony lid” OR “bony window”) AND (technique OR piezosurgery OR microsaw OR micro-saw OR “bone defect” OR “bone defects” OR “oral surgery” OR maxilla OR mandible OR “maxillary sinus” OR apicectomy OR “apical surgery” OR “endodontic surgery” OR “root-end surgery” OR implant OR implants OR “impacted teeth” OR “impacted tooth” OR “impacted molar” OR “impacted molars” OR “third molar” OR “third molars” OR “inferior alveolar nerve” OR “inferior alveolar nerves” OR cyst OR cysts OR “cystic lesion” OR “cystic lesions” OR “computer-guided” OR “osteoplastic procedure” OR “osteoplastic procedures” OR “sinus surgeries”) AND (LIMIT-TO (LANGUAGE, “English”))
Cochrane Central Register of Controlled Trials (CENTRAL)	(“bone lid” OR “bony lid” OR “bony window”) AND (technique OR piezosurgery OR microsaw OR micro-saw OR “bone defect” OR “bone defects” OR “oral surgery” OR maxilla OR mandible OR “maxillary sinus” OR apicectomy OR “apical surgery” OR “endodontic surgery” OR “root-end surgery” OR implant OR implants OR “impacted teeth” OR “impacted tooth” OR “impacted molar” OR “impacted molars” OR “third molar” OR “third molars” OR “inferior alveolar nerve” OR “inferior alveolar nerves” OR cyst OR cysts OR “cystic lesion” OR “cystic lesions” OR “computer-guided” OR “osteoplastic procedure” OR “osteoplastic procedures” OR “sinus surgeries”) in Title Abstract Keyword—in Trials

**Table 2:** Main reasons for exclusion after full-text screening.

Main Reasons for Exclusion	No.	References
Bone lids not repositioned	6	Choi et al., 2021 [35]; Hamdoon et al., 2021 [36]; Saibene et al. 2019 [40]; Bianchi et al., 2015 [33]; Bovi et al., 2010 [34]; Nordera et al., 2007 [39]
Technical note	3	Katauczek et al., 2015 [25]; Bacci et al., 2014 [13]; Yura et al., 2010 [44]
Case report or case series with *n* < 10 bone lids	12	Liu et al., 2021 [38]; Seo et al., 2021 [42]; Lee et al., 2020 [37]; Aliyev et al., 2019 [32]; Sukegawa et al., 2018 [24]; Chiapasco et al., 2017 [23]; Sivolella et al., 2015 [8]; Wang et al., 2015 [43]; Sohn et al., 2011 [45]; Degerliyurt et al., 2009 [7]; Scolozzi et al., 2009 [41]; Chiapasco et al., 2009 [16]

**Table 3:** Main features of the included studies.

Ref.	Study Design	Clinical Indication	Total No. of Pts.	Total No. of Bone Lid Pts.	Gender (M/F) *	Mean Age (Range), yr *	Jaw (Max /Mand)	No. of Cases (Bone Lid)	Mean Follow-Up (Range)	Bone-Cutting Instruments	Bone Lid Fixation
**Sukegawa et al., 2021** [46]	Retrospective cohort study	Radicular cyst; follicular cyst; benign tumor	30	30	19/11	36.7 (SD 19.6; range 12–77)	Max (11)/mand (16) ^§^	30	Max: > 1 yr; mand > 6 mths	Piezosurgery	Resorbable plates (PLLA, PLLA/PGA, u-HA/PLLA)
**Naros et al., 2019** [17]	Case series	Treatment of maxillary sinus fungus ball	22	21	12/10	58.3 (SD 14.7)	Max	21	12.9 mths	Thin cutting wheel; piezosurgery	Suture
**Ahmed et al., 2018** [47]	Prospective cohort study	Impacted mandibular third molar removal	18	18	7/11	24.5 (20–29)	Mand	18	6 mths	Disc microsaw (inferior cut) + reciprocating saw	None
**Hu et al., 2018** [26]	Case-control study	Odontogenic maxillary sinus cyst removal	45	22	27/18	43.26 (17–68)	Max	22	3 mths	Piezosurgery	Miniplates
**Kablan et al., 2017** [48]	Case series	Impacted mandibular third molar removal	9	7	5/4 (bone lid 3/4)	Total 30.7 (12–75); bone lid 20.3 (12–27)	Mand	10 (+2 not repositioned)	>1 yr	Small round bur (superior osteotomy) + disc microsaw	None (2); microplates (8)
**Sivolella et al., 2017** [4]	Case series	Cyst/keratocystic odontogenic tumor enucleation; impacted teeth extraction (with or without associated cysts); apicectomy	21	21	15/6	40.5 (18–72)	Mand	21	2.3 yrs (1–6 yrs)	Piezosurgery	Miniplates
**Hu et al., 2015** [49]	Case series	Displaced root fragment removal from sinus	21	10	9/12	43.4 (22–60)	Max	10	(3 mths–2.5 yrs)	Piezosurgery	Microplates
**Xu et al., 2015** [50]	Case series	Dentigerous cyst removal	20	20	11/9	35 (17–68)	Max	20	14 mths (6–24 mths)	Piezosurgery	Microplate
**Biglioli and Chiapasco, 2014** [14]	Case series	Displaced implant removal from sinus	36	36	19/17	50.8 (28–72)	Max	36	(4–6 mths)	Piezosurgery or reciprocating saw; diamond bur (for upper horizontal osteotomy)	Resorbable suture
**Pappalardo and Guarnieri, 2014** [2]	Randomized prospective study	Mandibular cyst enucleation	80	40	35/45 (bone lid 21/19)	Piezo group 43.2 (21–67); control group 41.4 (20–66)	Mand	40	1 wk	Piezosurgery	None
**Jung et al., 2013** [10]	Case series	Compromised implant removal/replacement	10	10	9/1	61.5 (47–89)	Max (4)/mand (6)	10	_	Disc microsaw; shank drill (in some cases in addition to microsaw)	None; microplate (1 case)
**Khoury, 2013** [9]	Prospective cohort study	Implant explantation; fractured teeth/roots removal; impacted tooth extraction; nerve decompression/lateralization; displaced implant/foreign body removal from sinus	200	200	62/138	55.3 (19–74)	Max/mand	200	≥4 years (192 cases)	Disc microsaw	None; suture (sinus); micro-screws
**Oh et al., 2012** [3]	Case-control study (Retrospective)	Mandibular cyst removal	60	34	22/38	38.5	Mand	34	13.7 mths (6–24 mths)	_	Microplates (14); wire (7); resorbable sutures (6); mix (2); none (5)
**Kurokawa et al., 2002** [51]	Case series	Treatment of odontogenic maxillary sinusitis	53	53	32/21	44.5 (20–73)	Max	53	2 yrs	Diamond disk	Resorbable suture (45); microplates (8)
**Choung and Choung, 1997** [52]	Case series	Treatment of maxillary sinusitis; oroantral fistula closure; cyst enucleation; treatment of osteomyelitis; removal of impacted tooth in the sinus	24	24	17/7	37 (16–66)	Max	25	>48 mths	Oscillating saw (round blades); bur (6 cases)	Resorbable suture (20); none (5)
**Choi et al., 1996** [53]	Randomized prospective study	Displaced tooth removal from sinus; treatment of chronic sinusitis with oroantral communication	20	20	8/12	46 (25–60)	Max	20	3 mths	Fissure bur and chisel	Resorbable suture
**Widmark et al., 1992** [54]	Case series	Treatment of maxillary sinusitis; cyst enucleation; displaced tooth removal from sinus; treatment of cases of pain in healthy sinus	12	12	6/6	45 (26–80)	Max	13	2 (1–4 yrs)	Small drills and osteotome	Resorbable suture
**Lasaridis et al., 1991** [55]	Case series	Apicectomy of mandibular molars	21	21	_	_	Mand	24	At least 6 mths	Round and fine fissure burs	None
**Khoury and Hensher, 1987** [1]	Case series	Apicectomy of mandibular molars	75	75	_	_	Mand	75	3–6 mths	Round bur and chisel	None; resorbable suture
**Lindorf, 1984** [56]	Case series	Rhinogenous cause; dentogenous cause; accident; treatment of tumors	61	61	_	_	Max	70	6 mths	Small circular saw	None; resorbable suture; fibrin-based adhesive

* If not specified, gender and age refer to all patients, including non-bone-lid; ^§^ 30 cases (i.e.,16 in the mandible, 11 in the maxilla, and 3 unspecified); mand: mandible; max: maxilla; mth(s): month(s); PGA: polyglycolic acid; PLLA: poly-L-lactic acid; u-HA: uncalcined and unsintered hydroxyapatite particles; wk(s): week(s); yr(s): year(s).

**Table 4:** Summary of the main results of studies related to different surgical applications.

Application	References	No. of Cases (Bone Lid)	Post-Op Assessment Methods	Main Findings
**Root-end surgery**	**Lasaridis et al., 1991** [55]	24	- Clinical evaluation - Radiographic assessment (intraoral X-ray)	Healing in resected roots: complete in 19 teeth, uncertain in 4 teeth (16.7%). **Complications:** unsatisfactory healing in 1 tooth (4.2%); transient paresthesia in 4 cases (resolved within 2 months).
	**Khoury and Hensher, 1987** [1]	75	- Clinical evaluation - Radiographic assessment (intraoral X-ray; panoramic X-ray)	Good healing after 3 months in all cases except one, and in the majority complete healing within 6 months. **Complications:** infection and removal of bone lid in a patient who did not take antibiotics (1 case); in some patients (number not specified) transient postoperative paresthesia resolved within 1 month.
**Access to mandibular lesions and impacted teeth**	**Sukegawa et al., 2021** [46]	16	- Clinical evaluation - Radiographic assessment (panoramic X-ray; CT scan)	Overall, 30 cases; i.e., 16 in the mandible, 11 in the maxilla, and 3 unclear localization (see “other indications”). Bone healing in 27 cases and bone lid necrosis in 3. No significant differences between patients with bone healing and bone lid necrosis as regards age, sex, anatomical variables (jaw, side, cortical bone thickness), lesion size, pathological diagnosis, and osteosynthesis material. Significant differences in smoking (*p* = 0.005), alcohol intake (*p* = 0.003), and in the distance of the lesion from the alveolar bone crest (*p* = 0.037); i.e., in cases of necrosis, the lesions were closer to the alveolar bone crest. **Complications:** 3 bone lid necrosis (out of 30 total cases): all in the mandible, all in male patients and in presence of smoking habit and alcohol consumption, and all in cases of follicular cyst removal. Average lesion size in cases of necrosis: mesiodistal diameter 13.57 mm (SD 1.03) and buccolingual diameter 11.96 mm (SD 2.15).
	**Ahmed et al., 2018** [47]	18	- Clinical evaluation - Radiographic assessment (CBCT)	Good intraoperative fit of the 3D-printed cutting guide in all patients. Average operative time: 25 min. Pts. showed normal parameters of pain, facial swelling, and maximal mouth opening. Uneventful primary wound healing in all pts.; no signs of infection, flap dehiscence, or bone exposure. Immediate post-op CBCTs showed proper repositioning of the bony segment; 6 months post-op CBCTs showed adequate cortication of the buccal plate of bone and normal bone healing of the sockets in all cases. **Complications:** Transient lower lip paresthesia in 3 pts., probably owing to pressure during root removal (recovery achieved within 3 months).
	**Kablan et al., 2017** [48]	10	- Clinical evaluation - Radiographic assessment (panoramic X-ray; CBCT)	Uneventful healing in all cases, but three; in 1 case the bone lid was not repositioned and used for anterior augmentation allowing implant placement in the recipient site. **Complications:** infection and second surgery for bilateral bone lid removal (in 2 cases in the same pt., in which the bone lids were not fixated); transient hypoesthesia completely resolved after 2 months (in 2 pts.)
	**Sivolella et al., 2017** [4]	21	- Clinical evaluation - Radiographic assessment (panoramic X-ray; CT scan)	Clinical follow-up 2 weeks post-op: soft tissues healed by primary intention, normal color, no inflammation, no signs of necrosis/suppuration/bone lid exposure. Miniplates were removed in 8 cases (2 for pt. discomfort, 3 for fistula, 3 for prosthetic reasons). Complete radiographic bone defect filling in 18 cases; radiographic integration of the bone lid in 20 cases (in the case of bone necrosis: revision surgery and removal of the necrotic lid); pre-/1 y post CT (11 cases): 93.8% mean reduction in volume of the radiolucent areas; no cyst recurrence. **Complications:** immediate complications: bleeding/edema (1); ecchymosis (1); IAN paresthesia (3: regressed spontaneously in 2 cases after 2 and 4 months; 1 permanent)—late complications: trismus (1); broken screws during miniplates removal (3); discomfort due to miniplates (2); fistula (2); IAN hypoesthesia (1); bone lid necrosis (1); persistent radiolucent lesion (2).
	**Pappalardo and Guarnieri, 2014** [2]	40	- Clinical evaluation - Radiographic assessment (panoramic X-ray, Dentalscan)	Piezosurgery group (bone lid) presented lower pain symptoms, minor swelling, and less recovery time compared to the conventional rotatory-group, and no lesions of mandible nerve. **Complications**: none in piezosurgery/bone lid group; 2 pts. in conventional group presented with paresthesia 1 week post-op.
	**Oh et al., 2012** [3]	34	- Clinical evaluation - Radiographic assessment (panoramic X-ray)	Correlation between bony healing, determined through panoramic radiographs, and surgical approach (bone lid or not repositioned) was not significant after 6, 12, and 24 months post-op (*p* <0.05). **Complications:** none in control group. In bone lid group, 12 pts. with complications: continuous discomfort disappeared after wire removal (2); dysgeusia disappeared after plate removal (1); infection and incision, drainage of abscess, and sequestrectomy (4); recurrent aspects observed and re-surgery performed (3); curettage since bone grafting failed (1); abnormality of bone lid location and removal (1).
**Implant explantation**	**Jung et al., 2013** [10]	10	- Clinical evaluation - Radiographic assessment (intraoral X-ray)	Successful implant removal and bone lid repositioning in all cases; in 3 cases, successful immediate implant placement. **Complications:** none.
	**Khoury, 2013** [9]	146	- Clinical evaluation - Radiographic assessment (intraoral X-ray; panoramic X-ray; CT scan)	Good bone healing in all cases but 3. **Complications:** in 3 pts., in which implants affected by peri-implantitis were explanted, resorption of the bone lid occurred (3 months post-op: in 1 pt. resorption >30%; in 2 pts. resorption >50%).
**Access to the maxillary sinus**	**Naros et al., 2019** [17]	21	- Clinical evaluation - Histopathologic examination (of fungus ball)	Complete removal of the fungus ball in all cases. **Complications:** recurrence after 3 years in 1 case (treated with revision surgery) and insufficient sinus ventilation in 2 cases (treated with revision infundibulotomy).
	**Hu et al., 2018** [26]	22	- Clinical evaluation - Radiographic assessment (CT scan)	Surgeries completed in 20 min. Perfect intra-operative anesthetic effect; small amount of intra-operative blood loss (except in 2 cases); complete removal of all lesions with sinus mucosa preservation; easy repositioning of bone lid, with the use of iodoform gauze due to excessive exudation in 2 cases; durations of pain and swelling (all cases without infraorbital involvement) 2–7 days (mean 3.62 days) and 5–14 days (mean 6.47 days) respectively; no bone resorption and no change in maxillary contour; only 2 cases had mild sinus mucosal thickening. **Complications:** in 2 cases, intraoperative impulsive bleeding (due to damage of the posterior superior alveolar artery); nasal bleeding (1–3 days) in 8 cases; post-op infection in 1 case (clinical symptoms disappeared after removing miniplate and bone lid and draining for 3 month); in 2 cases, mild sinus mucosal thickening.
	**Hu et al., 2015** [49]	10	- Clinical evaluation - Radiographic assessment (CT scan)	Surgical procedure was completed within 20 min. All patients healed without oroantral communications. Duration of pain was 3 to 14 days, and swelling was 2 days to 3 months. No pt. developed facial paresthesia or asymmetry. CT scans showed no lesions in the maxillary sinus and the morphology of alveolar bone was normal with little loss of height and width. **Complications:** 1 pt. experienced discharge and fullness after surgery (settled within 2 months).
	**Xu et al., 2015** [50]	20	- Clinical evaluation - Radiographic assessment (CT scan)	No need for further treatment except in one case; post-op pain and swelling mild or moderate (none severe); no facial paresthesia; no nasal obstruction; no hemorrhage; no recurrence. Post-op CT: sinus extrusion deformation improved to different degrees and cured chronic maxillary sinusitis. **Complications:** microplate removal and radical maxillary sinusotomy owing to infection (1 case).
	**Biglioli and Chiapasco, 2014** [14]	36	- Clinical evaluation - Radiographic assessment (panoramic X-ray, CT scan)	Displaced implants retrieved in all cases; surgical procedure <30 min; uneventful post-op recovery; in all pts. with pain before surgery, complete regression of symptoms; CT (4–6 months post-op): correct stabilization of bone lid, no significant bone lid resorption, and no gap between bone lid margins and surrounding bone; and in the 3 pts. presenting preoperatively with hypertrophic sinus mucosa, 1 complete regression, and in 2, some residual thickening, but with no signs and symptoms of sinusitis. In 12 pts., sinus grafting 12–18 months after bone lid in the same areas and 17 implants placed in the grafted areas 6–9 months later (implant survival rate 100%, and no complications). **Complications:** none.
	**Kurokawa et al., 2002** [51]	53	- Clinical evaluation - Radiographic assessment (CT scan)	No sensory impairment of skin and mucosa supplied by the infra-orbital nerve 2 years after surgery (in 4 pts., temporary loss of sensitivity). No loss of dental sensitivity 2 years after surgery (in 2 pts., temporary loss). Radiologically: reimplanted bone remained intact and no scar tissue invaded the maxillary sinus. The drainage window constructed in the lower nasal cavity remained patent. **Complications:** inflammatory complications in 2 pts. (1 pt. treated without the removal of the reimplanted bone, in 1 pt., removal of the reimplanted bone); 1 case of recurrent sinusitis (successfully treated with Caldwell-Luc method); temporary loss of sensitivity (skin, mucosa) in 4 cases; transient loss of dental sensitivity in 2 cases.
	**Choung and Choung, 1997** [52]	25	- Clinical evaluation - Radiographic assessment (panoramic X-ray; CT scan; bone scintigraphy; Water’s view)	Exact bone lid repositioning; adequate vascularity of the bone lid; no neurosensory disturbances; normal tooth sensitivity. **Complications:** facial pain disappeared within 1 month after surgery (1 case).
	**Choi et al., 1996** [53]	20	- Radiographic assessment (CT scan)	Group A: bone lid as a free bone graft (10 cases); Group B: osteoperiosteal pedicled bone lid (10 cases). Bone lid consolidation: observed in all Group B cases, partial loss of bone lid in 2 cases of Group A. Bone density: significant differences in bone density between the 2 groups (*p* < 0.05), mean bone reduction 55% in group A, while no reduction in group B; no significant difference in bone density between pts. with and without chronic maxillary sinusitis. **Complications:** partial loss of bone lid in 2 cases of Group A.
	**Widmark et al., 1992** [54]	13	- Clinical evaluation - Radiographic assessment (sinus radiography; CT scan)	Surgical procedure uneventful in 10 cases out of 12 (in 2 cases, bone lid fragmentation); normal tooth sensitivity; bone lid integration and healthy sinus in most cases. **Complications:** bone lid fragmentation during surgery (2 cases). Infraorbital nerve injury: 10 days post-op hypoesthesia (8 cases) and paresthesia (1 case)—3 months post-op hypoesthesia (5 cases)—1-year post-op hypoesthesia (3 cases). Radiographic findings (CT): 3 months post-op mild dislocation of the bone lid (1 case) and suspicious of fracture (1 case); 1 year after surgery in 2 cases almost complete opacification of the sinus; in 1 (out of 4) long-term follow-up, pt. had thickened sinus mucosa.
	**Lindorf, 1984** [56]	70	- Clinical evaluation - Endoscopic evaluation - Radiographic assessment	A total of 96% of the cases of chronic or sub-acute sinusitis were cured by the first surgical treatment; no loss of dental sensitivity; 3 out of 6 pts with residual problems were cured by appropriate medications. **Complications:** 3 pts. with residual problems were surgically treated again (1 case of new formation of hemangiomatous fibroma + 2 cases of polypoid sinusitis).
**Other indications**	**Sukegawa et al., 2021** [46]	14	- Clinical evaluation - Radiographic assessment (panoramic X-ray; CT scan)	Access to alveolar bone lesions: 11 in the maxilla and 3 unclear locations. For details, see above. **Complications:** all in the mandible, none in the maxilla.
	**Khoury, 2013** [9]	47	- Clinical evaluation - Radiographic assessment	Fractured tooth/root and foreign body removal (29 cases) + impacted tooth extraction (14 cases) + inferior alveolar nerve decompression—lateralization (4 cases) Uneventful healing after all procedures. **Complications:** none.

**Table 5:** Risk of bias assessment: reviewers’ judgments about each risk of bias item for each included RCT.

References	D1	D2	D3	D4	D5	D6	D7	Overall
**Pappalardo & Guarnieri, 2014** [2]	Moderate	Low	Moderate	Low	Low	Low	Moderate	Moderate
**Choi et al., 1996** [53]	Low	Low	Moderate	Moderate	Low	Low	Moderate	Moderate

Low = all criteria were met, and no more than one criterion was judged unclear. Moderate = two or more criteria were judged unclear, and the other criteria were met. Domains: D1, random sequence generation (selection bias); D2, allocation concealment (selection bias); D3, blinding of participants and personnel (performance bias); D4, blinding of outcome assessment (detection bias); D5, incomplete outcome data (attrition bias); D6, selective reporting (reporting bias); D7, other bias (e.g., sample size calculation).

**Table 6:** Risk of bias assessment: reviewers’ judgments about each risk of bias item for each included observational study.

References	Selection of Participants	Sample Size ^§^	Follow-Up ^§§^	Drop-Outs ^§§§^	Measurement of Outcome	Selection of Reported Result	Overall
**Sukegawa et al., 2021** [46]	Low	Moderate	Moderate	Low	Low	Low	Moderate
**Naros et al., 2019** [17]	Low	Moderate	Moderate	Low	Low	Low	Moderate
**Ahmed et al., 2018** [47]	Low	Moderate	Low	Low	Low	Low	Low
**Hu et al., 2018** [26]	Low	Moderate	High	Low	Low	Low	High
**Kablan et al., 2017** [48]	Low	High	High	Low	Low	Low	High
**Sivolella et al., 2017** [4]	Low	Moderate	Moderate	Low	Low	Low	Moderate
**Hu et al., 2015** [49]	Low	Moderate	Moderate	Low	Low	Low	Moderate
**Xu et al., 2015** [50]	Low	Moderate	Moderate	Low	Low	Low	Moderate
**Biglioli and Chiapasco, 2014** [14]	Low	Moderate	Moderate	Moderate	Low	Low	Moderate
**Jung et al., 2013** [10]	Low	Moderate	Low	Low	Low	Low	Low
**Khoury, 2013** [9]	Low	Low	Low	Low	Low	Low	Low
**Oh et al., 2012** [3]	Low	Moderate	Moderate	Low	Low	Low	Moderate
**Kurokawa et al., 2002** [51]	Low	Low	Moderate	High	Low	Low	High
**Choung and Choung, 1997** [52]	Low	Moderate	High	Moderate	Low	Low	High
**Widmark et al., 1992** [54]	Low	Moderate	High	Low	Low	Low	High
**Lasaridis et al., 1991** [55]	Low	Moderate	Moderate	Low	Low	Low	Moderate
**Khoury and Hensher, 1987** [1]	Low	High	Moderate	Low	Low	Low	High
**Lindorf, 1984** [56]	Low	Low	Moderate	Low	Low	Low	Low

Low = all criteria were met, and no more than one criterion was judged unclear. Moderate = two or more criteria were judged unclear, and the other criteria were met. High = one or more criteria were not met. § Low >50, medium 11–50, high <10; §§ low >5 yrs, medium 1–5 yrs, high <1 yr; §§§ low <5%, medium 5–15%, high >15%.

## Data Availability

Data will be provided upon reasonable request.
